# Optimal Duration of Monitoring for Atrial Fibrillation in Cryptogenic Stroke: A Nonsystematic Review

**DOI:** 10.1155/2016/5704963

**Published:** 2016-05-29

**Authors:** Essa Hariri, Ahmad Hachem, Georges Sarkis, Samer Nasr

**Affiliations:** ^1^School of Medicine, Lebanese American University, P.O. Box 36, Byblos, Lebanon; ^2^Department of Pediatrics and Adolescent Medicine, American University of Beirut Medical Center, 6th Floor, P.O. Box 11-0236, Riad El-Solh, Beirut 1107 2020, Lebanon; ^3^Faculty of Medicine, Lebanese University, P.O. Box 6573/14, Badaro, Museum, Beirut, Lebanon; ^4^Department of Cardiology, Mount Lebanon Hospital-Gharios Medical Center, P.O. Box 470, Camil Chamoun Boulevard, Hazmieh, Lebanon

## Abstract

Atrial fibrillation (AF) is the most common form of cardiac arrhythmias and an independent risk factor for stroke. Despite major advances in monitoring strategies, clinicians tend to miss the diagnoses of AF and especially paroxysmal AF due mainly to its asymptomatic presentation and the rather limited duration dedicated for monitoring for AF after a stroke, which is 24 hours as per the current recommended guidelines. Hence, determining the optimal duration of monitoring for paroxysmal atrial fibrillation after acute ischemic stroke remains a matter of debate. Multiple trials were published in regard to this matter using both invasive and noninvasive monitoring strategies for different monitoring periods. The data provided by these trials showcase strong evidence suggesting a longer monitoring strategy beyond 24 hours is associated with higher detection rates of AF, with the higher percentage of patients detected consequently receiving proper secondary stroke prevention with anticoagulation and thus justifying the cost-effectiveness of such measures. Overall, we thus conclude that increasing the monitoring duration for AF after a cryptogenic stroke to at least 72 hours will indeed enhance the detection rates, but the cost-effectiveness of this monitoring strategy compared to longer monitoring durations is yet to be established.

## 1. Introduction

Atrial fibrillation is the most common form of arrhythmias with prevalence increasing from 0.5% in the 50–59 age group to more than 8% in the 80–89 age group [1]. Atrial fibrillation (AF) puts the patient at an increased risk of stroke. Strokes from AF are generally severe in their outcomes and more disabling [2]. This fact is moreover complicated by the common silent nature of atrial fibrillation as approximately one-third of the patients affected are asymptomatic [3]. However, silent AF is associated with at least the same risk for stroke compared to symptomatic AF. Paroxysmal atrial fibrillation (PAF) is as much implicated in stroke risk as permanent atrial fibrillation [4]. It is estimated that between 25 and 60% of atrial fibrillations are paroxysmal in nature [5].

Atrial fibrillation is the most common cause of stroke in the elderly population [5]. Despite the many technological advances in regard to stroke care and prevention, almost 30% of all strokes remain without an identifiable cause after extensive workup and are thus referred to as “cryptogenic” [6]. Current evidence based medicine guidelines recommend treating such patients with antiplatelet therapy [7]. However, this may be insufficient when the source is embolic in nature. In the latter case, oral anticoagulation (OAC) using vitamin K antagonists or novel oral anticoagulants (NOACs) must be added to the patient's regimen to prevent any further embolic events as strokes from AF tend be disabling. The recurrence rate of stroke is as high as 30% [8], mostly during the first year, and holds higher mortality rates. Occult PAF seems to be one of the culprits of “cryptogenic” strokes, particularly in the elderly. This is especially likely for patients with an “embolic pattern” of ischemia on neuroimaging (i.e., involvement of multiple vascular territories seen on MRI diffusion restriction), which may be seen in 12% of cryptogenic events [9]. Such patients are prime targets for extended duration electrocardiogram (ECG) monitoring.

One American Heart Association (AHA) guideline states that cardiac monitoring “should be conducted routinely after an acute cerebrovascular event to screen for serious cardiac arrhythmias” [10]. The latest AHA guidelines recommend at least 24 hours of continuous cardiac monitoring for the early management of patients with stroke for detection of PAF [7]. However, little consensus has been reached regarding the optimal method and duration of monitoring. This review aims to discuss the different evolving monitoring strategies of paroxysmal atrial fibrillation following a cryptogenic stroke and highlight the benefits of prolonged monitoring beyond the 24-hour timeline recommended at the present time, particularly with 72 hours of continuous ECG monitoring (CEM).

## 2. Atrial Fibrillation and Stroke

AF is the leading cause of stroke among patients > 75 years old and is responsible for at least 15% of all strokes [11]. AF-induced strokes are associated with greater morbidity and mortality and more severe neurological deficits (National Institutes of Health Stroke Scale >10) when compared to strokes not associated with AF [12]. The former are largely avoidable with the use of oral anticoagulants, which provide an additional 40% reduction of stroke risk compared to monotherapy with antiplatelets [13] and together with the management of hypertension can prevent more than 65% of all strokes [14]. However, the current evidence shows that patients with cryptogenic stroke or transient ischemic attack (TIA) are generally treated with antiplatelet therapy and control of vascular risk factors [7], which although sufficient for arterial sources of embolism tends to undertreat embolism of cardiac origin. Hence, it is essential to detect the presence of AF after a stroke to maximize secondary prevention. Current standard of care includes admission, 12-lead electrocardiogram (ECG), and a workup of thrombotic causes of stroke that includes CT scan, MRI scan, carotid duplex scan, followed by echocardiography, and at least 24 hours of CEM [15]. However, the optimal use of these diagnostic techniques to guide the proper therapeutic intervention and secondary prevention remains a topic of debate.

Several studies have investigated a correlation between the incidence of AF and the type of stroke. AF is usually more prevalent in patients with cryptogenic stroke compared to those with large atherothrombotic and lacunar strokes [16], but this is not a consistent finding [17]. Moreover, the incidence of AF is higher in patients with anterior circulation territory infarction when compared to those with lacunar strokes (68% versus 0%). Moreover, multiple lesions (embolic pattern) are a feature of long-lasting AF rather than paroxysmal AF [18]. Older age is the most common risk factor consistently associated with higher risk of AF due to accumulation of vascular risk factors in these patients, but additional findings such as frequent premature atrial complexes ECG/Holter [19–21] and left atrial dilatation with reduced left atrial appendage ejection fraction on transthoracic and transesophageal echocardiogram can help stratify the stroke risk further [21–24]. All in all, a combination of clinical and radiological features (comprising embolic infarct pattern, age >65 years, and preexistent coronary artery disease) has been associated with a higher incidence of AF [25]. Based on our literature review, prolonged ECG monitoring should be considered in all stroke patients without known AF, including patients with stroke of known causes such as carotid stenosis or small-artery disease [26].

## 3. Paroxysmal Atrial Fibrillation and Stroke

### 3.1. Definition and Risks of PAF

Paroxysmal atrial fibrillation is not clearly defined in the literature, with multiple studies using different definitions to classify supraventricular tachyarrhythmias (SVT) as paroxysmal, detected with different types of ECG monitors. Yet, most studies defined PAF as events lasting more than 30 seconds, derived from the AHA 2006 guidelines [10]. PAF is a self-promoting process that can progress to persistent AF if left undetected and untreated with rhythm control and anticoagulation [27]. In AF, diminished blood flow within the complex trabeculated anatomy of the left atrial appendage (LAA) promotes thrombus formation with associated inflammation, leading to stroke by embolization [28]. However, it is still poorly understood whether PAF leads to thrombus formation in the LAA and its subsequent dislodgement during periods of sinus rhythm leading to stroke.

A new classification nomenclature is recently described regarding types of strokes called “Embolic Strokes of Undetermined Source,” or ESUS, which aims to provide new therapeutic dimensions in the management of stroke [29]. By definition, a new stroke is defined as ESUS if it is a nonlacunar brain infarct detected after ruling out the presence of extracranial or intracranial atherosclerosis (causing ≥50% luminal stenosis in arteries supplying the ischemic area) and a major cardioembolic source. Under such nomenclature, paroxysmal atrial fibrillation is identified as the most frequent cause (42.9%) of ESUS [29].

Nevertheless, it has been shown that PAF is more prevalent than persistent AF in patients with CVA [3]; one large prospective study identifies two-thirds of cases of AF as paroxysmal [30]. Another prospective study compares the rates of PAF in patients with stoke of known cause versus cryptogenic stroke [31]. The study found that PAF is more common in cryptogenic stroke than in stroke of known cause in patients younger than 65 years of age (22% versus 3%; *p* = 0.07), but detection rates were similar in older patients. Hence, PAF may not be the only explanation behind an episode of cryptogenic stroke.

Furthermore, the risk of stroke in patients with PAF determined by standard surface ECG tracings is similar to that observed with chronic and persistent forms of AF [4], and thus these patients are eligible to stroke prophylaxis in a manner identical to persistent forms [10, 32]. Besides, the contribution of PAF to the risk of stroke can also be seen in trials involving patients with implanted cardiac devices that monitor episodes of atrial tachyarrhythmias. In the Mode Selection Trial (MOST), the presence of atrial high-rate episodes (>220 bpm) lasting >5 minutes in a cohort of 312 patients with intracardiac therapeutic devices was associated with 6.7-fold increased risk of stroke and a 2.48-fold increased risk of death [33]. The TRENDS trial was a prospective observational study of a cohort of 2486 patients with pacemakers or cardioverter-defibrillators. In this cohort, a threshold of atrial tachyarrhythmia/AF burden > 5.5 hours with atrial rate > 175 bpm lasting ≥ 20 seconds during a day on any of the preceding 30 days of monitoring was associated with doubling of the risk of thromboembolism [34]. The Asymptomatic Atrial Fibrillation and Stroke Evaluation in Pacemaker Patients and the Atrial Fibrillation Reduction Atrial Pacing Trial (ASSERT) evaluated 2580 patients ≥65 years of age with hypertension and an implanted pacemaker or defibrillator but no previously documented AF. The presence of occult tachyarrhythmias (defined as 190 bpm for >6 minutes) during the first 3 months of monitoring was associated with 2.5-fold increase in the risk for ischemic stroke and systemic embolism [35]. In addition, AF and paroxysmal AF are found to be associated with a higher risk for “silent” stroke, a subtype of stroke detected only by brain MRI [36, 37].

### 3.2. Diagnosis and Current Trends in Monitoring

A major problem with PAF is that most episodes are asymptomatic, a fact confirmed by multiple studies and trials. The Suppression of Paroxysmal Atrial Tachyarrhythmias (SOPAT) trial [68] showed that only 46% of telephonic electrocardiograms recorded during the 1-year follow-up period were associated with specific symptoms. Similarly, the Prevention of Atrial Fibrillation After Cardioversion (PAFAC) trial [69] revealed that, of the 191,103 recordings using daily telephonic ECG monitoring, 70% of all AF were asymptomatic. Studies done with implantable cardiac devices have shown that more than 90% of stored atrial arrhythmia episodes were asymptomatic [70, 71]. A study by Page et al. [72] also demonstrated the lack of correlation between symptoms and the presence of PAF, where patients were likely to experience 12 episodes of asymptomatic AF for every episode of PAF.

This brings us to the question of what duration of atrial fibrillation events will be associated with a future risk of stroke. Literature does not show any clear association between the duration of AF events and stroke risk, yet it has been shown that higher burden of AF events (i.e., total duration) is associated with higher stroke risk. A total AF burden higher than 5.5 hours is predictive of future stroke, extrapolated from a long duration of monitoring (>365 days) [34]. Similarly, a recent study showed that patients with subclinical atrial tachyarrhythmias of >6 minutes have increased rates of AF as well as ischemic stroke events [35].

Not only is the definition of PAF debatable, but also the detection methods of these atrial high-rate episodes vary greatly between studies. Since the discovery by Dr. Holter in the early 1960s of a method to record, store, and display cardiac electric waves [73], different cardiac devices with more sophisticated designs and technology of storage and analysis have become available to monitor and detect asymptomatic PAF and guide appropriate therapeutic interventions [74]. The different methods of monitoring, their pros and cons, and their detection sensitivity and specificity are discussed in detail in recent review articles [1]. Basically, prolonged cardiac monitoring includes noninvasive techniques such as surface ECG recorders on one hand and invasive methods that rely on subcutaneous recording systems or intracardiac recording systems. Given the asymptomatic nature of PAF, patient-activated devices are of low clinical value, and monitoring devices identify episodes of PAF automatically based on specific algorithms of ECG analysis that vary across devices. Currently, available monitors have several limitations. Holter monitoring, although sensitive and specific for PAF detection, is limited by the short duration of monitoring (1-2 days in most practices). Moreover, devices relying on skin contact electrodes can cause skin irritations, making it difficult for patients to wear such devices for long durations. One example is the external loop recorders, which can be worn for extended periods of time but rely on patient's recognition of palpitations (active patient recording), thus incurring the probability of missing asymptomatic PAF. Another monitoring system is ambulatory telemetry, which has a battery-powered sensor with a capacity of ≤30 days of ECG data storage and high sensitivity to PAF episodes (as short as a few seconds). Its most prominent disadvantages are the high cost and low patient compliance. To limit skin irritation and noncompliance, implantable loop recorders were developed and are usually placed subcutaneously using a minimally invasive procedure. Monitoring for ≤3 years, MRI compatibility, and wireless transmission to the physician are available features, yet only episodes of arrhythmia ≥2 minutes can be detected. Finally, dual-chamber pacemakers and implantable cardioverter-defibrillators are intracardiac devices that can be programmed to detect atrial tachyarrhythmias and act accordingly to maintain a normal rate. However, these devices are of limited therapeutic value and indicated only in patients with life-threatening arrhythmias [1]. Finally, although ambulatory ECG monitoring was found to be superior continuous inpatient ECG [25], contradicting data showed superiority of continuous inpatient ECG over ambulatory ECG monitoring [49]. Hence, no consensus exists regarding the perfect cardiac monitoring method to detect AF.

On the other hand, several studies have evaluated the significance of clinical, radiologic, and echocardiographic factors that could be potential predictors of the presence of PAF after cryptogenic stroke, which can facilitate risk stratification of these patients and selection of suitable candidates for evaluation by CEM. Predictors of PAF include diabetes, female gender, premature atrial complexes (PACs), slow heart rhythm on ECG, left atrial dilatation, left ventricular reduced ejection fraction (EF) on transthoracic echocardiogram (TTE), higher stroke severity assessed by National Institute of Health Stroke Scale (NIHSS), cortical infarcts on neuroimaging, and congestive heart failure [9, 20, 43, 62, 75]. Such predictors may identify which patient is more likely to have PAF after a cryptogenic stroke and subsequently may be eligible for CEM. Given the high cost of CEM, this would be a more cost-efficient option. As a result, multiple risk assessment scoring schemes have been developed for prediction of PAF [76, 77], including the score for the targeting of AF (STAF) [78] and the recently developed iPAB score (identified by past history of arrhythmia or antiarrhythmic agent use, atrial dilation, and BNP elevation), which was the first prospective multicentered cohort analysis of predictive score for PAF after ischemic stroke, proving superior to all other validated scores [79]. Besides, biomarkers such as elevated B-type natriuretic peptide (BNP) (cut-off = 35 pg/mL) [80] and elevated troponin I [81] are associated with a higher likelihood of PAF. Finally, high CHADS_2_ and CHA_2_DS_2_-VASc scores, but not baseline ischemic stroke, predict new-onset AF in a 10-year follow-up on ischemic stroke patients [12]. Despite the validated sensitivity and specificity of these scoring systems [82], a physician must not forget the clinical judgment when selecting candidate patients for long-term monitoring.

## 4. Optimal Duration of Screening for Atrial Fibrillation after Stroke

### 4.1. Continuous ECG Monitoring: Current Evidence

Although patients in common practice receive a single 24- or 48-hour Holter monitor after a cryptogenic stroke as per the current guidelines for stroke management [15], the diagnosis of PAF is very often missed, and unidentified patients will thus miss on anticoagulation with better secondary stroke prevention. Patients will certainly benefit from continuous electrocardiographic monitoring (CEM) after stroke based on observations from clinical trials in patients with recurrent AF [69, 72], several single-center studies, and studies on implantable cardiac defibrillators or pacemakers [35]. These studies have used a variety of cardiac monitors for PAF detection, with a detection yield ranging from 0% to 25% months [5, 9, 16–18, 20, 21, 25, 41–43, 45, 47–50, 52–58, 60–67, 83, 84] (Table 1). In a recent meta-analysis by Sposato et al. (2015) investigating the frequency of detected PAF after ischemic stroke among 50 studies (with 11,658 patients), the proportion of patients with poststroke AF was 7.7% for those in the emergency room, 5.1% in those who worn serial ECGs, 10.7% in those using Holter monitor as the first period of ambulatory monitoring, and 16.9% in those using mobile cardiac outpatient telemetry, external loop recording, and implantable loop recording as a second ambulatory [11]. The overall detection yield of AF after all phases of cardiac monitoring was 23.7%.

One trial that utilized CEM was the Cryptogenic Stroke and Underlying Atrial Fibrillation trial (CRYSTAL AF). In this trial, patients with cryptogenic stroke were randomly assigned to either conventional follow-up (control) or CEM with an insertable cardiac monitor (ICM, Reveal XT; Medtronic, Minneapolis, Minnesota). The rate of AF detection was 8.9%, 12.4%, and 30% in the CEM arm versus 1.4%, 2.0%, and 3.0% in the control arm, at 6, 12, and 36 months, respectively (*p* < 0.001 for all) [6]. A recent study involving 168 patients with ICM of the CRYSTAL AF trial also showed that, for a single monitoring period, the sensitivity of AF detection with intermittent monitoring was lowest with a 24-hour Holter (1.3%) and highest with a 30-day event recorder (22.8%) [84]. However, long-term monitoring remained significantly superior in detecting AF compared to intermittent monitoring. Besides, the 30-Day Cardiac Event Monitor Belt for Recording Atrial Fibrillation After a Cerebral Ischemic Event (EMBRACE) trial showed that AF was detected in 16.1% in the intervention arm using a 30-day event-triggered recorder compared to only 3.2% in the control arm with conventional 24-hour monitor [63]. Overall, the average incidence of AF reported in a systematic review by Seet et al. [1] done on a total of 3039 subjects was 6.3%, the highest incidence being in patients older than 60 years using continuous inpatient ECG and 24-hour Holter monitoring (21.3%) [52]. However, the use of implantable loop recorders was not able to detect new cases of AF in younger patients with cryptogenic stroke. It is important to mention that most of these studies are single-center surveys, with a retrospective design in which the investigators employed investigational devices such as invasive implantation of ECG monitors. On the other hand, studies using external monitors worn for 24 to 72 hours reported a relatively good AF detection rate of AF 2.4% to 6.0% [18, 41].

In addition, studies with implantable cardiac devices vary widely in their design, methods of monitoring, study population, stroke characteristics, intervals of monitoring, and most importantly the definition of PAF. Furthermore, and in the absence of control groups, these studies were not instrumental in changing the current recommendation for AF diagnosis (24 hours of Holter ECG) and their conclusions did not make it into clinical practice. For instance, the EMRACE-STEMI trial had used specific loop recorder currently as part of their trial design that is not available at most clinical sites, thus making it difficult to implement the investigators' recommendations at the present time [85]. Regarding the therapeutic value of implanted cardiac devices with long-term monitoring, the IMPACT trial evaluated whether AF detection using these devices will guide the use of anticoagulation and subsequently decrease the risk of stroke [86]. The trial included 2718 patients randomized to either dual-chamber and biventricular defibrillators (with decision on anticoagulation based on remote rhythm monitoring) or the usual office-based follow-up (with anticoagulation determined by standard clinical criteria). The trial results did not show any difference in rates of stroke, systemic embolism, or major bleeding and henceforth the trial was stopped after 2 years. Hence, the strategy of early initiation or halting of anticoagulation based on AF detection using remote implanted cardiac devices did not prevent thromboembolism and bleeding.

### 4.2. Optimal Duration of Monitoring for PAF/AF after Stroke

Evidently, it is still unclear what the optimal duration of such cardiac monitoring is given that the cost-effectiveness of prolonged ECG monitoring has been questioned [87]. Despite the fact that more prolonged cardiac monitoring increases the detection rate of PAF after cryptogenic stroke, the question remains whether currently available forms of external monitoring systems can substitute these invasive and costly continuous monitoring devices. In the latest guidelines for stroke prevention, there is weak evidence suggesting prolonged rhythm monitoring for AF for around 30 days within 6 months of an ischemic stroke/TIA (class IIa, level of evidence C) [7].

Multiple studies have investigated the outcome of extending the current recommended 24-hour monitoring to 72 hours in terms of AF detection yield. A multicenter cohort study by Grond et al. (2013) compared 72- and 24-hour monitoring standard Holter ECG monitoring for detection of unknown AF, applied on admitted patients who are survivors of previous CVA [61]. Using 72-hour ECG monitoring, unknown AF was detected in 49 out of 1135 patients (4.3%, 95% confidence interval, 3.4–5.2%), while it was detected only in 29 patients (2.6%) within the first 24 hours of ECG monitoring. Another recent prospective study by Lee et al. (2015) showed the efficacy and importance of CEM for at least 3 days, which had an overall PAF detection rate of 8% and 10% in nonlacunar strokes and with doubling of the detection rate of AF [65]. However, there was no difference in the detection rates between those with cryptogenic stroke versus stroke of known cause. In another study, the incidence of AF was 6% using 72-hour ambulatory ECG monitoring device among patients with suspected embolic stroke [41]. Similarly, studies by Douen et al. [44] and Schuchert et al. [41] suggest that 72 hours of monitoring for AF after a stoke is a superior monitoring tool to its 24-hour counterpart. Besides, serial ECG assessments within the first 72 hours of an acute stroke significantly improve detection of AF by 2.6-fold compared to 24-hour Holter monitoring [3]. Another study by Suissa et al. (2013) showed that using CEM increases the detection yield of AF after a stroke by 5.29-fold compared to the routine 24-hour Holter ECG, when adjusted to potential confounders (demographic data, vascular risk factors, and National Institutes of Health Stroke Scale scores). Additionally, the study showed that, beyond 3 days, the usefulness of CEM compared to the routine strategy lost significance and thus concluded that early detection is optimal and decreases beyond 72 hours [23]. These findings are further consolidated by a study by Rizos et al. [53], which reveals that the maximum detection of new AF cases (50%) was seen in the first 36 hours after admission to the stroke unit, which is consistent with other reports showing a maximum detection rate of AF occurring in the first 3 days of hospitalization [49, 55]. Last but not least, one pilot randomized clinical trial of 40 patients studying the effect of prolonged monitoring on therapeutic directions of stroke patients showed no benefit of long-term monitoring over routine clinical follow-up [59]. Evidently, CEM with a 72-hour strategy is a feasible method in most healthcare settings, given that patients with stroke are admitted for at least 3 days for workup; hence it can be easily implemented without interfering with medical management. And when it comes to cost-effectiveness of 72-hour monitoring, Grond et al. [61] reported a similar detection rate (4.3%) compared to study by Kamel et al. [87] that proved the cost-effectiveness of 1-week outpatient monitoring using a wide range of model inputs in sensitivity analyses, with a cost flexibility depending on the drug regimens used [88]. Hence, with a lower duration of monitoring and similar detection rate, there will be a decrease in cost-per-gain in quality-adjusted life-years. Overall, although extending ECG monitoring for 72 hours undoubtedly increases the detection yield of new AF compared to the standard 24 hours of monitoring (Table 1), the issue of cost-effectiveness of this strategy compared to long-term ECG monitoring beyond 72 hours is yet to be established.

## 5. Conclusion and Future Directions

The importance of cardiac monitoring for AF after a cryptogenic stroke has been well documented. A significant proportion of these patients are found to have AF after monitoring, and detecting this arrhythmia is crucial to giving these stroke survivors the benefits of secondary prevention provided by anticoagulation therapy. Different monitoring techniques have been utilized in different studies on PAF detection, and advances in methods of cardiac monitoring, from outpatient monitoring to invasive cardiac implants, have made it possible to increase the yield of PAF documentation that was previously underdiagnosed. Although the current stroke guidelines recommend only at least 24 hours of Holter ECG monitoring, extending this duration with continuous ECG monitoring has been shown by multiple studies to substantially increase the chances of detecting these silent tachyarrhythmia episodes (Table 1). However, the several flaws in the design of these studies and the weak level of evidence they provide rendered the implementation of their recommendations in daily clinical practice difficult. This delayed long-awaited guidelines updates until stronger evidence emerged. Future studies should therefore focus on finding the optimal duration of monitoring for AF following an acute ischemic stroke while at the same time determining the most significant predictors of AF to select the best candidates for monitoring and the most appropriate and cost-effective monitoring strategy.

Regarding ongoing research in this field, the Find-AF_RANDOMISED_ is a recently ongoing randomized and controlled prospective multicenter trial of 400 patients ≥60 years with ischemic stroke to be randomized for either prolonged ECG monitoring (10 days at baseline and after 3 and 6 months) or regular standard of care (≥24-hour continuous ECG monitoring) and with at least 12-month follow-up. This trial will be the first randomized controlled multicenter trial using prolonged Holter ECG monitoring with current standard-of-care procedures in an unspecific stroke population, and results are expected to be released by spring 2016. This trial will provide valuable information relevant to the ongoing debate of the optimal monitoring duration for AF after a stroke. Another ongoing clinical trial is the Impact of Standardized Monitoring for Detection of Atrial Fibrillation in Ischemic Stroke (MonDAFIS) trial, a prospective randomized multicenter study with 3,470 patients who had an acute ischemic stroke or transient ischemic attack and without known AF on hospital admission [89]. This study is the largest to date aiming at evaluating the impact of prolonged and systematic ECG monitoring during hospital stay (up to 7 days in hospital) on secondary stroke prevention compared to usual stroke unit diagnostic procedures for detection of AF (control group). By analyzing the proportion of patients receiving anticoagulation at 12 months, this study will certainly help in guiding therapeutic interventions for patients with cryptogenic stroke based on CEM, which is still an understudied area of stroke management.

Based on this review, we have shown that the optimal duration of monitoring for AF is yet to be determined. Besides, and in light of the current evidence, monitoring for at least 72 hours should be performed in all patients with cryptogenic stroke as a feasible monitoring strategy that is warranted to improve patient outcomes due to enhanced detection rates. However, the optimal duration pertaining to cost-effectiveness is yet to be determined, given the potential for further prevention of stroke recurrence with higher detection rates as a result of monitoring strategies longer than 3 days. Eventually, further well-controlled studies and randomized clinical trials that tackle the cost-effectiveness of long-term monitoring will hopefully provide the answer to how long a patient should be monitored for AF after a cryptogenic stroke.

## Figures and Tables

**Table 1:** Detection rates of atrial fibrillation after stroke using different monitoring strategies.

Authors	Publication year	Method of recording	Sample size	Approximate recording duration (hour)	AF detection rate (%)
Rem et al. [38]	1985	CICT	184	72	3.5
Koudstaal et al. [39]	1986	Holter	100	24	5
Hornig et al. [40]	1996	Holter	300	24	3
Schuchert et al. [41]	1999	Holter	82	24	1
Schuchert et al. [41]	1999	Holter	82	72	6
Barthélémy et al. [17]	2003	Holter	60	24	10
Barthélémy et al. [17]	2003	Holter	28	96	14.3
Barthélémy et al. [17]	2003	ELR	60	70	15
Jabaudon et al. [18]	2004	Holter	169	24	5.7
Schaer et al. [42]	2004	Holter	425	24	2
Shafqat et al. [16]	2004	Holter	465	24	2.4
Jabaudon et al. [18]	2004	ELR	88	1320	5.7
Wallmann et al. [19]	2007	ELR	127	504	14
Tayal et al. [43]	2008	MCOT	56	504	23
Douen et al. [44]	2008	Holter	144	72	10.4
Vivanco Hidalgo et al. [45]	2009	CICT	465	55	3.4
Schaer et al. [46]	2009	Holter	241	24	0
Elijovich et al. [47]	2009	CICT	218	48	1
Elijovich et al. [47]	2009	MCOT	20	720	20
Yu et al. [48]	2009	Holter	96	24	9.4
Gaillard et al. [20]	2010	TTM	98	720	9.2
Alhadramy et al. [5]	2010	Holter	413	24	2.7
Lazzaro et al. [25]	2012	Holter	133	30	6
Lazzaro et al. [25]	2012	CICT	133	74	0
Rizos et al. [49]	2010	CICT + Holter	136	48 + 24	21.3
Stahrenberg et al. [50]	2010	Holter	224	168	12.7
Ziegler et al. [51]	2010	ILR (ICD)	163	9360	28
Dion et al. [52]	2010	ILR	24	10440	0
Bhatt et al. [9]	2011	MCOT	62	504	24
Rizos et al. [53]	2012	CEM	496	64	8
Kallmünzer et al. [54]	2012	CEM	501	72	16
Sposato et al. [55]	2012	CICT	155	480	14
Flint et al. [56]	2012	MCOT	239	720	12.1
Ritter et al. [57]	2013	Holter	60	168	2
Higgins et al. [58]	2013	Holter	50	336	8
Kamel et al. [59]	2013	MCOT	50	504	0
Cotter et al. [21]	2013	ILR	54	1152	26
Ritter et al. [57]	2013	ILR	60	1536	17
Etgen et al. [60]	2013	ILR	393	8760	27
Grond et al. [61]	2013	Holter	1135	72	4.3
Miller et al. [62]	2013	Holter	156	504	17.3
Gladstone et al. [63]	2014	ELR	572	720	16.1
Brambatti et al. [64]	2014	ILR (ICD)	261	2.160	10
Lee and Sun [65]	2015	CEM	395	72	7
Yayehd et al. [66]	2015	SFM	56	504	1.7
Brachmann et al. [67]	2016	ILR	221	25920	30

AF, atrial fibrillation; CEM, continuous stroke unit ECG monitoring; CI, confidence interval; CICT, Continuous Inpatient Cardiac Telemetry; ELR, external loop recorder; ICD, implantable cardiac defibrillator; ILR, Internal Loop Recorder; MCOT, mobile cardiac outpatient telemetry; SFM, Spider Flash Monitor.
